# The Transactions of the Hom. Med. Society of New York, 1883

**Published:** 1883-10

**Authors:** 


THE TRANSACTIONS OF THE HOMŒOPATHIC MEDICAL SOCIETY OF THE STATE OF NEW YORK. 1883.

This volume contains the proceedings of the thirty-first and thirty-second semi-annual meetings of the Society. There are many very interesting papers reported. New York has a large number of able homœopathists, and this volume contains some of their work. We believe the Secretary, Dr. A. P. Hallet, has a few copies for sale.

